# A Case of Uterine Broad Ligament Hernia Difficult to Distinguish From Adnexal Torsion

**DOI:** 10.7759/cureus.78790

**Published:** 2025-02-09

**Authors:** Kazuaki Shima, Masakazu Nishida, Kentaro Kai, Kosuke Suzuki, Eiji Kobayashi

**Affiliations:** 1 Department of Obstetrics and Gynecology, Nakatsu Municipal Hospital, Nakatsu, JPN; 2 Department of Obstetrics and Gynecology, Faculty of Medicine, Oita University, Yufu, JPN; 3 Department of Gastroenterology Surgery, Usuki Cosmos Hospital, Usuki, JPN

**Keywords:** acute abdomen, adnexal torsion, intestinal obstruction, pelvic surgery, uterine broad ligament hernia

## Abstract

Uterine broad ligament hernia is a rare type of internal hernia, with symptoms similar to those of adnexal torsion. Distinguishing these disorders is crucial. We treated a 49-year-old Japanese woman with a history of gravida (n=5), parity (n=3), and cesarean sections (n=2). After visiting a physician due to abdominal pain and vomiting, she was referred to our hospital based on suspicion of ovarian torsion. Emergency surgery at our hospital revealed that the patient's small intestine was contained within the defective hilar membrane, resulting in a uterine broad ligament hernia. With the cooperation of the intestinal surgeon, we performed a resection and anastomosis of the necrotic small intestine in the herniated area. The patient's postoperative course was uneventful, and she was discharged on the 9th postoperative day. A uterine broad ligament hernia causes necrosis of the small intestine and can be life-threatening. Women with a history of surgery or delivery might be at higher risk for this hernia. The swollen small intestine at the hernia site is often mistakenly diagnosed as adnexal torsion. In this patient's case, we diagnosed the uterine broad ligament hernia with necrotic small intestine during the surgery. The prompt surgery enabled a good patient prognosis.

## Introduction

A uterine broad ligament hernia is a female-onset internal hernia caused by an abnormal defect and weakness of the uterine broad ligament, accounting for 4%-5% of all internal hernias [1]. The preoperative diagnosis of this disorder is challenging due in part to its rarity compared to hernias such as mesenteric defect hernia. Adnexal torsion is a more common emergency condition among gynecological disorders, and it must be differentiated from rare uterine broad ligament hernia. Obstruction of the blood flow to the intestinal tract and adnexa causes irreversible necrosis, and these disorders thus require immediate decompression surgery, especially in cases that include intestinal necrosis, which is life-threatening and requires an even faster response.

We describe the case of a patient with a uterine broad ligament hernia that was difficult to distinguish from adnexal torsion.

## Case presentation

The patient was a 49-year-old Japanese premenopausal woman, gravida 5, parity 3. She had undergone an ovarian cystectomy twice. This patient had a left ovarian cystectomy at age 34 and a left oophorectomy at age 40, as well as three previous deliveries. More recently, she consulted her primary care physician about a sudden onset of abdominal pain and vomiting. At the hospital, a CT scan suggested possible adnexal torsion due to an ovarian tumor or a swollen fallopian tube, and she was referred to our hospital for emergency care.

**Figure 1:**
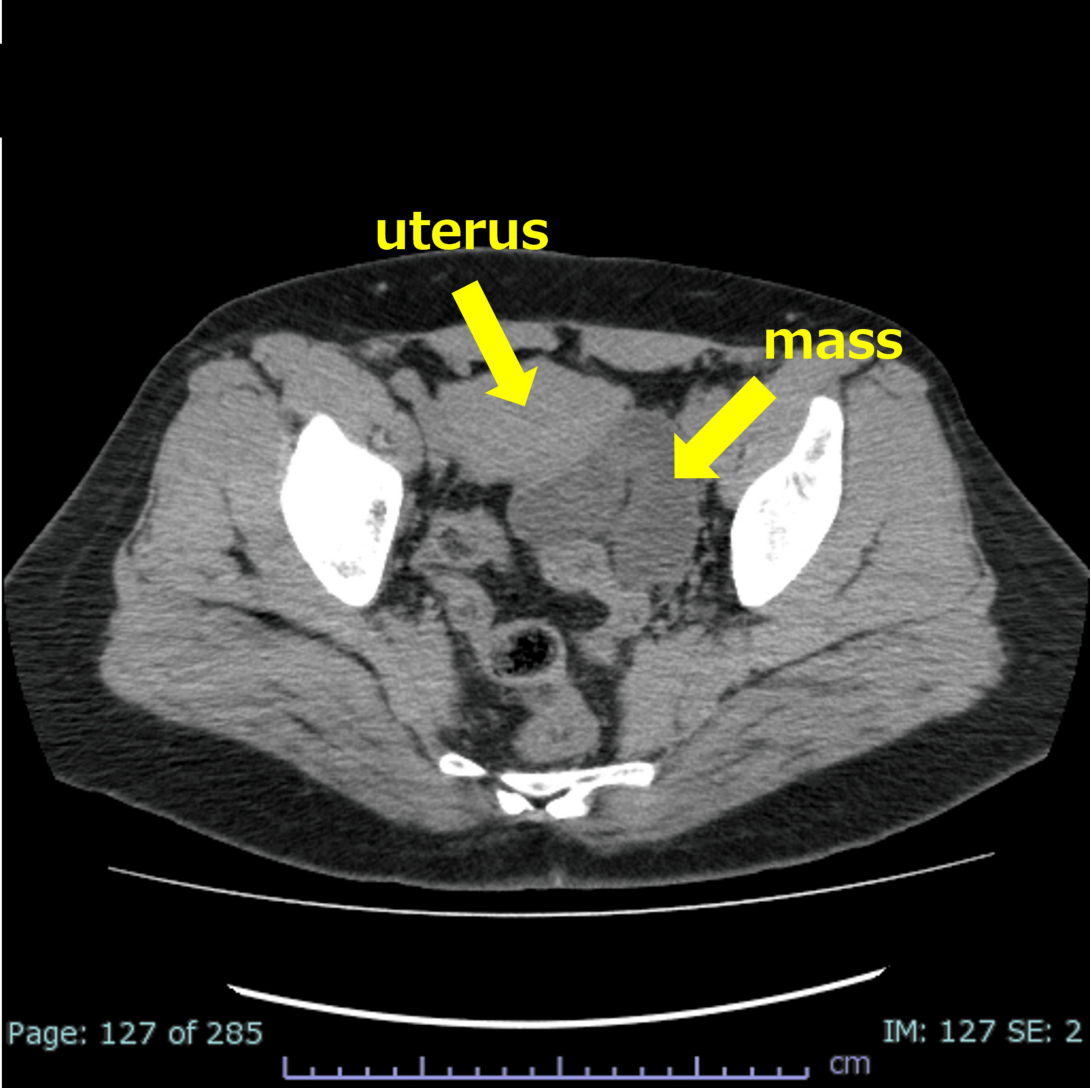
CT scan findings. The patient's CT scan showed a sausage-shaped enlarged mass on the left posterior of her uterus, which was suspected to be a torsion of the fallopian tubes based on the imaging results.

On admission, she was 164 cm tall, weighed 80 kg, and showed a temperature of 36.9°C, blood pressure of 153/94 mmHg, heart rate of 59 beats/min, and oxygen saturation (SpO2) of 99% (room air). She exhibited tenderness throughout the lower abdomen but no muscular defense. Blood tests showed a white blood cell count at 9,790/mm^3^, neutrophil ratio at 88.0%, Hb at 11.7 g/dL, and C-reactive protein (CRP) at 0.02 mg/dL. A CT examination revealed a tortuous left tubular mass in the pelvic cavity behind the uterus (Figure 1). We diagnosed adnexal torsion based on the CT scan findings and planned a laparoscopic surgery.

**Figure 2:**
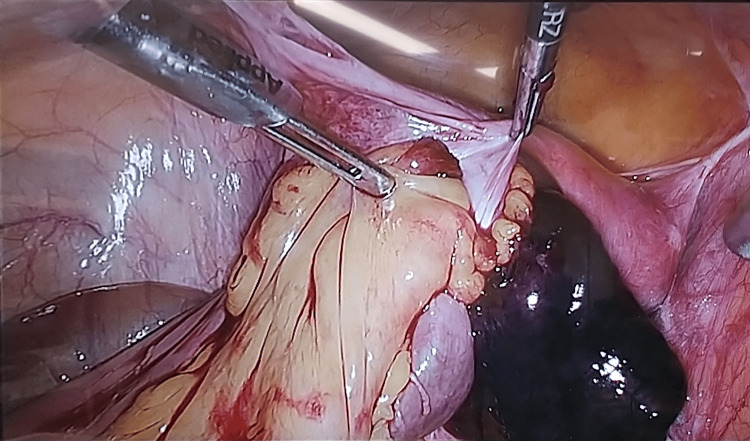
Intraoperative findings with necrotic intestinal tract. The small intestine was located within a deficient uterine broad mesentery. Its blood flow had ceased, and it was partially necrotic.

**Figure 3:**
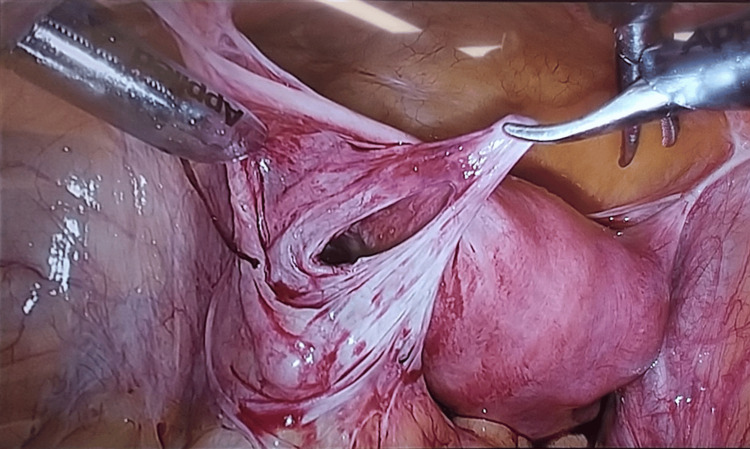
Intraoperative findings after bowel resection. Hernia sacs are found in the area of a uterine broad ligament.

The surgical findings included reddish-black discoloration and necrosis of the intestinal tract (Figure 2). During the surgery, we observed that the small intestine with a black color was fitted into an abnormal defect in the posterior left uterine broad ligament. After the herniated small intestine was released, the cord-like material that formed the hernia portal was removed (Figure 3). 

**Figure 4:**
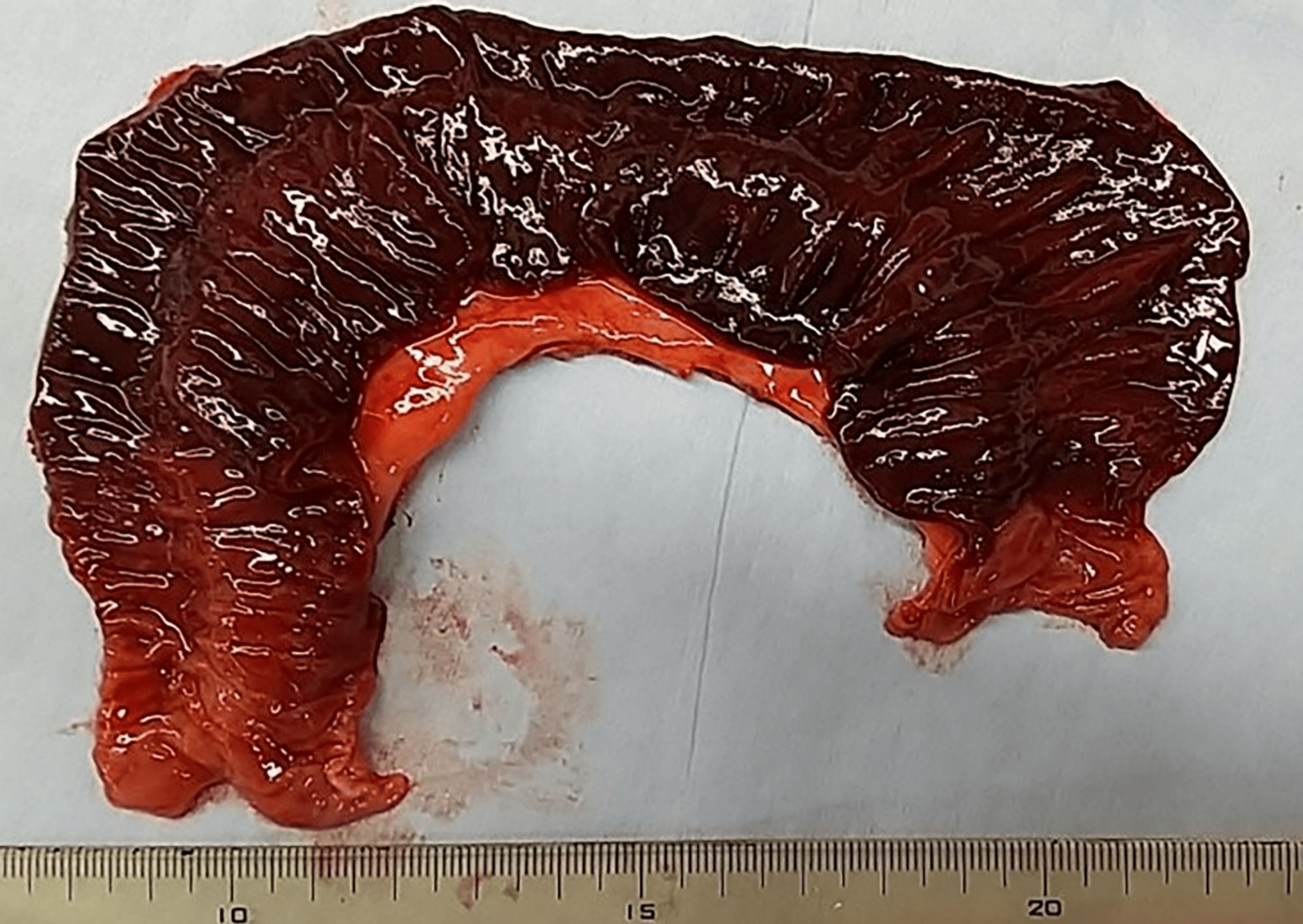
Findings of resected intestinal tract. Small intestine with extensive necrosis is seen.

The subsequent resection and reconstruction of the necrotic small intestine were performed by the intestinal surgeon (Figure 4). In this surgery, the herniated small intestine was first removed. Since part of the small intestine was necrotic, this part was resected and re-sutured by the gastrointestinal surgeon. The hernia portal was closed with continuous sutures to prevent recurrence. The patient's postoperative course was uneventful. The patient began walking the day after surgery, had a bowel gas on the third postoperative day, and resumed eating. The patient was on a regular diet on the sixth postoperative day and was discharged on the ninth postoperative day without any complications. The results of the pathology examination were the foundation of the diagnosis of ischemic small bowel inflammation.

## Discussion

Internal hernias are very rare, with a frequency of 0.2%-0.9%; the present patient's hernia, i.e., a uterine broad ligament hernia, is a type of internal hernia and is reported to occur in 4%-7% of all cases of internal hernias [2,3]. Table 1 describes the characteristics of this type of hernia with reference to the relevant literature, including the reports by Sajan et al. and Takahashi et al. [2,4]. According to those authors, the herniated organ is most often the small intestine, rarely the ovary or sigmoid colon, but in some cases both. There is no significant difference in the laterality of the hernia, and most of the patients have a history of surgery or delivery.

**Table 1:** Characteristics of a uterine broad ligament hernia. Each item and number in this table is based on the tables in the papers by Sajan et al. and Takahashi et al. [2,4]. N/A: not available.

Hernia organs	Small bowel	50
	Sigmoid colon	4
	Ovary	2
	Sigmoid colon+ovary+fallopian tube	1
	Transverse colon+small bowel	1
	Fallopian tube+small bowel	1
	Fallopian tube	0
	None	1
	Total	60
Defected location	Left	19
	Right	19
	Bilateral	9
	N/A	2
	Total	49
Surgical history	Appendectomy	5
	Cesarean section	8
	Other (including multiple previous surgeries)	15
	Vaginal delivery	28
	N/A	4
	Total	60
Diagnostic images	CT scan	16
	Ultrasound-sonography	4
	X-ray	1
	Other than the above (including combinations of the above)	27
	N/A	1
	Total	49
Intervention	Laparoscopic surgery	52
	Open	8
	Total	60

Among the various examinations, CT imaging is the most effective for evaluating blood flow disorders in the herniated organs, and early surgery is necessary if a blood flow disorder of the intestine is suspected. Surgery has been the treatment of choice in many cases, and 90% of the surgeries have been performed with laparoscopy [5,6]. Atileh et al. reported that the treatment for a uterine broad ligament hernia does not require using mesh and are treated only with sutures of the hernia sac [6]. Moreover, Mazzetti et al. reported that (i) the age of onset of a uterine broad ligament hernia has been the late 30s to 40s, (ii) the patients had a history of two or more deliveries, and (iii) they had the symptoms of abdominal pain, nausea, and vomiting as the main complaints in common [7].

It is said that the causes of uterine broad ligament hernias include congenital anomalies, external forces such as childbirth, labor, and surgery, tissue adhesions and deviations due to pelvic infections, and decreased elasticity of the uterine broad ligament due to aging [8]. Our patient had a history of five abdominal surgeries, including two ovarian cystectomies and three cesarean sections, which might be additional risk factors for uterine broad ligament hernia. In contrast, adnexal torsion, which is common disorder in the gynecological field, can be caused by an enlarged ovary or a swollen fallopian tube. Especially in cases of fallopian tube obstruction, the enlarged fallopian tube resembles the intestinal tract. In our patient's case, the CT examination showed no change in the density of the fatty tissue around the mass (which is a finding of intestinal obstruction), and the tumor lumen was homogeneous, and thus intestinal hernia was ruled out before the patient's surgery. In light of the tumor's shape, the patient's disorder was diagnosed as torsion of a fallopian tube, and emergency laparoscopic surgery was performed. Based on the intraoperative findings, it was diagnosed as a uterine broad ligament hernia, and partial necrosis of the intestine was observed.

Because of the preoperative consultation, we were able to respond quickly after the intraoperative identification of intussusception and necrosis due to the patient's uterine broad ligament hernia, and since ileus is often identified by CT scan and treated, there are many reports of uterine broad ligament hernias by gastroenterological surgeons and physicians. In addition, in our patient's case, four departments' physician were involved in the diagnosis before the surgery, but a definitive diagnosis was not reached until the surgery was performed. If a gynecological disorder such as ovarian torsion is suspected, surgery is usually chosen by the patient's gynecologist alone, but if the disorder is unexpected, e.g., intestinal necrosis, close cooperation among specialists in several fields is necessary. Clinicians' familiarity with and recognition of these disorders may also contribute to the diagnosis of rare diseases.

## Conclusions

A uterine broad ligament hernia has symptoms similar to those of uterine adnexal torsion, but as in the present case, this hernia is sometimes accompanied by necrosis of the intestinal tract and thus requires prompt surgery. The diagnosis may be made intraoperatively, and it is important to establish a trusting relationship with other specialists in order to perform the surgery smoothly in cooperation with other hospital departments. We hope that this case report will raise awareness of uterine broad ligament hernias, increase the possibility of its preoperative diagnosis, and contribute to improving the quality of medical care in the field of emergency abdominal diseases.
